# Ruminal Fermentation, Methanogenic Archaeal Diversity, and Metabolomic Responses to *Urochloa brizantha* Extracts in Sheep

**DOI:** 10.3390/metabo16090641

**Published:** 2026-09-02

**Authors:** Janaina Silveira da Silva, Rafaela Scalise Xavier de Freitas, Jaqueline Fernandes Bruno, Guilherme Pegoraro Rissi, Eduardo Solano Pina dos Santos, Vinicius Laerte Silva Herreira, Nara Regina Brandão Cônsolo, Gabriel Henrique Ribeiro, Luiz Alberto Colnago, Ives Cláudio da Silva Bueno

**Affiliations:** 1 Department of Animal Science, College of Animal Science and Food Engineering, University of São Paulo (FZEA/USP), Pirassununga 13.635-900, SP, Brazil; scaliserafaela@gmail.com (R.S.X.d.F.); jaquelinebruno@usp.br (J.F.B.); guilherme.rissi@usp.br (G.P.R.); ivesbueno@usp.br (I.C.d.S.B.); 2 Department of Animal Nutrition and Production, School of Veterinary Medicine and Animal Science, University of São Paulo (FMVZ/USP), Pirassununga 13.635-900, SP, Brazil; eduardosolano@usp.br (E.S.P.d.S.); vinicius.laerte@usp.br (V.L.S.H.);; 3 Brazilian Agricultural Research Corporation (EMBRAPA) Instrumentation, São Carlos 13.561-206, SP, Brazilluiz.colnago@embrapa.br (L.A.C.); 4 Department of Chemistry, Federal University of São Carlos, São Carlos 13.565-905, SP, Brazil

**Keywords:** phytogenic additives, rumen modulators, ethanolic extract, hydroethanolic extract, untargeted metabolomics, ovine

## Abstract

Background: Phytogenic feed additives are being explored as alternatives to conventional rumen modulators, yet their effects on the ruminal ecosystem remain insufficiently characterized when assessed only through conventional fermentation parameters. This study evaluated the effects of ethanolic and hydroethanolic extracts of *Urochloa brizantha* on ruminal fermentation, methanogenic archaeal diversity, and the ruminal metabolome of sheep. Methods: Eight rumen-cannulated wethers were assigned to a replicated 4 × 4 Latin square design with four 28-day periods. Treatments consisted of no additive (CTL), ethanolic extract of *U. brizantha* (EE; 50 mL/day), hydroethanolic extract (HE; 80 mL/day), and monensin (MON; 25 mg/kg of DMI). Ruminal fluid was evaluated for fermentation traits, archaeal 16S rRNA gene diversity, and untargeted metabolomic profiles. Results: Dry matter intake and fermentation parameters were unaffected by treatment. Archaeal alpha diversity showed a contrast-dependent response, with lower Shannon diversity in sheep receiving plant extracts than in those receiving monensin (*p* = 0.034), and *Methanimicrococcus* was selectively enriched in the EE group (*p* = 0.012). Untargeted metabolomics revealed the clearest response: despite substantial overlap in global PCA profiles, PLS-DA indicated treatment-specific temporal separation between day 0 and day 21 only in EE-treated sheep, supported by permutation testing based on classification error rate (*p* = 0.027), although predictive performance should be interpreted cautiously. This EE-specific response was characterized by a significant shift in the carboxylic acid subclass (FDR = 0.022). Top VIP-ranked discriminants (aspartate, succinate, glutamate, phenylalanine, leucine, and choline) decreased nominally but failed multiple-testing correction (FDR = 0.0602–0.2580). CTL, HE, or MON showed no temporal separation. Conclusions: *Urochloa brizantha* extracts produced limited effects on conventional ruminal fermentation parameters but were associated with selective archaeal responses. In particular, the ethanolic extract was associated with a treatment-specific temporal metabolomic pattern, including a significant shift in the carboxylic acid subclass, while individual metabolite changes remained exploratory and warrant further investigation.

## 1. Introduction

Ruminant livestock play a crucial role in global food systems because they convert fibrous biomass into high-value animal products. However, this biological advantage is accompanied by inefficiencies inherent to ruminal fermentation, including the loss of dietary energy as enteric methane, which also contributes substantially to greenhouse gas emissions [1]. For this reason, improving ruminal efficiency has become a major objective in animal nutrition and sustainable livestock research.

Among the strategies developed for this purpose, ionophores such as monensin have been extensively used as feed additives to improve energy efficiency by shifting fermentation pathways toward greater propionate and, in some cases, lower methane output [2,3]. Although ionophores operate through specific ion-transport mechanisms and are distinct from medically important human antibiotics, growing consumer demand for natural livestock production and increasing global regulatory scrutiny regarding in-feed antimicrobial additives have intensified the search for plant-based alternatives, commonly known as phytogenic additives [4]. These additives contain plant secondary metabolites, such as saponins and lipophilic derivatives, which can potentially modulate the ruminal microbiome and alter fermentation profiles [5,6].

Despite growing interest in phytogenic feed additives as natural alternatives for modulating rumen fermentation, their mechanisms of action in the rumen are not yet fully understood [7]. Some studies suggest that plant-derived compounds can modulate ruminal fermentation and microbial populations, although responses often vary according to dose, botanical origin, and phytochemical composition [8]. One important source of this variability is that the biological activity of plant extracts depends strongly on their phytochemical composition, which is influenced by the extraction procedure, particularly solvent polarity [9]. Hydroethanolic solvents generally recover a broader range of polar and amphipathic compounds, whereas ethanol may preferentially enrich more lipophilic fractions [10]. Consequently, the choice of extraction method may determine not only the classes of compounds recovered, but also the magnitude and direction of their biological effects in the rumen.

However, many in vivo studies still assess phytogenic additives mainly through conventional fermentation endpoints, such as ruminal pH, ammonia nitrogen (NH_3_-N), and total short-chain fatty acids (SCFAs), which may not fully capture functionally relevant shifts in microbial ecology and metabolism. Because the ruminal microbiome exhibits substantial functional redundancy, biologically meaningful changes may occur even when these conventional parameters show limited variation [11]. In this context, untargeted metabolomics provides the biochemical resolution needed to uncover these hidden alterations. Therefore, an integrative approach is needed to understand how chemically distinct plant extracts affect specific microbial groups and metabolic pathways within the ruminal ecosystem [12].

In this context, *Urochloa brizantha*, widely cultivated forage species in Brazil [13], represents a promising source of bioactive compounds for ruminal modulation. Species within the *Urochloa* complex have been reported to contain steroidal saponins, including protodioscin, which have been associated with relevant biological effects in ruminants [14,15,16]. In addition, phytochemical screenings of *U. brizantha* extracts have identified other classes of compounds, such as alkaloids, steroids/triterpenoids, flavonoids, tannins, and saponins, depending on the extraction solvent [17]. Previous in vitro studies from our group showed that *U. brizantha* extracts can modulate ruminal fermentation [18]. However, their effects on the ruminal ecosystem remain largely unexplored in vivo.

Therefore, this study aimed to compare these extracts with monensin and the control diet by evaluating ruminal fermentation parameters (pH, NH_3_-N, SCFA), methanogenic archaeal diversity, and ruminal metabolomic profiles in sheep. We hypothesized that ethanolic and hydroethanolic extracts of *U. brizantha*, representing distinct phytochemical preparations and daily bioactive delivery levels, would differentially affect ruminal fermentation, archaeal community structure, and metabolomic responses.

## 2. Materials and Methods

Animal feeding management and sample collection were conducted at the School of Animal Science and Food Engineering of the University of São Paulo, located on the Fernando Costa Campus in Pirassununga, São Paulo, Brazil. The experimental protocol was approved by the Ethics Committee on Animal Use (CEUA) under approval number 1344101121.

### 2.1. Animal, Experimental Design, Treatments, and Diet

Eight rumen-cannulated wethers (Santa Inês × Dorper; 41.1 ± 8.1 kg BW) were allocated to a replicated 4 × 4 Latin square design (four treatments, four periods, two squares). Each 28-day period included baseline sampling on day 0, 21 days of additive supplementation (days 1 to 21), and 7 days of washout (days 22 to 28) before the subsequent period. The four treatments were: control (CTL, no additives), ethanolic extract (EE, 50 mL/day), hydroethanolic extract (HE, 80 mL/day), and monensin (MON, 25 mg/kg of DMI).

The *U. brizantha* cv. Marandu extracts were obtained using pilot-scale pressurized liquid extraction (PLE) at 80 °C and 10.35 MPa, following the agronomic and processing protocols previously described by [18]. The ethanolic extract (EE) was produced using 99.5% absolute ethanol, whereas the hydroethanolic extract (HE) utilized a 70% ethanol solution. Following PLE, HE was maintained in its original liquid state, whereas EE underwent controlled thermal reduction in a forced-air oven at 38 °C to lower residual ethanol concentration without degrading heat-sensitive bioactives. Protodioscin concentration was quantified via high-performance liquid chromatography (HPLC) [19] directly on the final formulations administered in vivo as 7.50 and 7.48 mg protodioscin/mL for EE and HE, respectively. Extracts were administered twice daily directly into the rumen via cannula using dosing syringes. To minimize the risk of ethanol-induced ruminal disruption, the daily volume of EE was capped at 50 mL/day (supplying 375.0 mg protodioscin/day), whereas HE was administered at 80 mL/day (supplying 598.4 mg protodioscin/day). Because daily protodioscin dose and extraction solvent co-vary, EE and HE represent two distinct extract treatment regimens supplying different bioactive delivery levels rather than an isolated solvent comparison.

The diet was formulated according to the National Research Council’s Nutrient Requirements of Small Ruminants (NRC) recommendations [20] to meet the maintenance requirements of sheep and was offered twice daily at 07:00 and 13:00 h. The forage-to-concentrate ratio was 70:30, with corn silage as the sole forage source. The experimental diet comprised 700 g/kg dry matter (DM) corn silage, 189 g/kg DM ground corn, 93 g/kg DM soybean meal, 6 g/kg DM limestone, and 12 g/kg DM mineral supplement. Its chemical composition included 436.1 g/kg DM (ID 930.15), 944.4 g/kg organic matter (OM), 55.6 g/kg ash (ID 942.05), 114.9 g/kg crude protein (CP; ID 954.01), 29.1 g/kg ether extract (ID 920.39), 438.6 g/kg neutral detergent fiber (NDF; ID 973.18), 245.1 g/kg acid detergent fiber (ADF; ID 973.18), 193.5 g/kg hemicellulose, and 361.8 g/kg non-fibrous carbohydrates (NFC), according to [21]. The mineral supplement provided (per kg) 130 g Ca, 65 g P, 130 g Na, 12 g S, 10 g Mg, 56,200 IU vitamin A, 7025 IU vitamin D3, 312 IU vitamin E, and trace minerals (48 mg Co; 148 mg Cu; 76 mg I; 3200 mg Mn; 18 mg Se; 3200 mg Zn; maximum 650 mg F).

Feed offered and refusals were recorded daily to determine dry matter intake (DMI), allowing for approximately 10% orts. Daily DMI values were averaged per animal within each experimental period prior to statistical analysis. Animals had free access to fresh water and mineral supplementation throughout the experiment.

On day 21 of each experimental period, rumen fluid was collected manually 4 h after morning feeding as a standardized post-feeding sampling time through the ruminal cannula from three different locations (cranial, central and caudal), pooled into a composite sample per animal per period, immediately flash-frozen in liquid nitrogen, and aliquoted for subsequent analyses of fermentation parameters, archaeal 16S rRNA gene sequencing, and metabolomic analyses. This sampling time was selected to ensure consistency across experimental periods and to synchronize the collection of samples for the integrated fermentation, archaeal, and metabolomic analyses. For untargeted metabolomics, additional samples were collected on day 0 (prior to the daily additive administration) to serve as period-specific baseline controls.

### 2.2. pH, Ammonia Nitrogen (NH_3_-N) and Short-Chain Fatty Acid (SCFA) Analysis

Rumen fluid was filtered through two layers of cheesecloth, and pH was measured immediately after collection using a digital bench pH meter (model W38, Bel Engineering^®^, Monza, Italy), calibrated with pH 4.0 and 7.0 buffer solutions. For NH_3_-N determination, rumen fluid was acidified with 1 N sulfuric acid (H_2_SO_4_), centrifuged (14,500× *g* for 10 min), and analyzed via distillation with 50% potassium hydroxide (KOH) followed by titration with 0.05 N H_2_SO_4_, according to the micro-Kjeldahl method of [22] and adapted by [23].

For SCFA analysis, samples were centrifuged (14,500× *g* for 10 min), and the supernatant was mixed with formic acid (98–100%) and an internal standard (100 mM 2-ethylbutyric acid, ChemService, West Chester, PA, USA). SCFA profiles were determined using gas chromatography (GC-2014, Shimadzu, Kyoto, Japan) equipped with a Stabilwax^®^ capillary column (Restek Corporation, Bellefonte, PA, USA) and a flame ionization detector [24]. Helium was used as the carrier gas, and concentrations were quantified using external standard calibration curves (ChemService, West Chester, PA, USA).

### 2.3. DNA Extraction and Archaeal 16S rRNA Gene Amplification

Total genomic DNA was extracted from pooled liquid and solid ruminal fractions from each animal-period sample (*n* = 32), using the PureLink™ Microbiome DNA Purification Kit (Invitrogen, Thermo Fisher Scientific, Waltham, MA, USA) according to the manufacturer’s protocol, including mechanical lysis to ensure efficient recovery of archaeal DNA. DNA integrity was assessed by agarose gel electrophoresis, and DNA concentration and purity were evaluated using a NanoDrop One spectrophotometer (Thermo Fisher Scientific, Waltham, MA, USA).

The hypervariable V8 region of the 16S rRNA gene was amplified, generating an approximately 315 bp amplicon, using primers 1106F (5′-TTWAGTCAGGCAACGAGC-3′) and 1378R (5′-TGTGCAAGGAGCAGGGAC-3′), primarily targeting methanogenic archaeal 16S rRNA genes, as described by [25]. Although targeting this single hypervariable region provides high specificity for methanogenic archaea, this short single-region amplicon assay inherently limits taxonomic resolution compared to full-length 16S rRNA gene sequencing or shotgun metagenomic approaches. Library preparation followed the Illumina 16S Metagenomic Sequencing Library Preparation protocol, including dual PCR amplification, bead purification (MagBio HighPrep PCR, MagBio Genomics, Inc., Gaithersburg, MD, USA), indexing with the Nextera XT v2 kit (Illumina, Inc., San Diego, CA, USA), and equimolar pooling of libraries. Sequencing was performed on an Illumina iSeq platform using paired-end 2 × 150 bp chemistry. PhiX control was spiked into the pooled library prior to sequencing. Samples collected at both baseline (day 0) and post-supplementation (day 21) were processed for archaeal sequencing, yielding 64 libraries in total. Following the loss of one sample during library preparation and/or quality filtering, 63 samples remained for downstream sequencing analyses.

Raw sequences were demultiplexed in BaseSpace^®^ (Illumina, San Diego, CA, USA) and quality-checked using FastQC. Downstream processing was conducted in R (v4.4.1) using the DADA2 package (v1.24.0). After primer removal, forward reads were filtered using the parameters trimLeft = 19, maxN = 0, maxEE = 2, and truncQ = 2, with PhiX removal enabled (rm.phix = TRUE). No fixed truncation length was imposed. Error rates were learned with learnErrors(), and ASVs were inferred using pooled sample inference (pool = TRUE). As the final ASV table was generated from denoised forward reads only, paired-end merging was not applied. Chimeric sequences were removed using removeBimeraDenovo() with the consensus method. Taxonomic classification was assigned against the SILVA database version 138.1 [26]. Alpha diversity was evaluated using Chao1 richness and the Shannon and Simpson diversity indices.

### 2.4. Sample Preparation and ^1^H NMR Metabolomics

Rumen fluid samples were ultrafiltered using 3 kDa centrifugal filters at 14,000× *g* for 30 min at 4 °C, and the resulting filtrate was used for ^1^H nuclear magnetic resonance (^1^H NMR) analysis. ^1^H NMR spectra were acquired at 298 K on a Bruker 600 MHz spectrometer (14.1 T, Bruker Corporation, Billerica, MA, USA) fitted with a 5 mm TCI cryogenic probe. One-dimensional ^1^H experiments were performed using the standard Bruker pulse sequence noesypr1d, which incorporates water signal suppression by continuous-wave presaturation. The acquisition parameters were set as follows: 128 scans, a relaxation delay of 4 s, a spectral width of 30 ppm, 4 dummy scans, an acquisition time of 3.635 s, and a 90° pulse duration of 9.75 μs. The data were processed by applying an exponential window function with 1 Hz line broadening and zero-filling to 256 k points prior to Fourier transformation. Spectra were processed using Chenomx NMR Suite Professional v7.7 (Chenomx Inc., Edmonton, AB, Canada). Baseline and phase corrections were performed individually for each spectrum, and pH adjustment was performed using imidazole resonances. Spectra were referenced to the methyl peak of 4,4-dimethyl-4-silapentane-1-sulfonic acid (DSS) at 0.00 ppm. DSS served as both the quantification reference and an internal standard, given its known concentration of 0.5 mM in each sample. Metabolite quantification was conducted using the Profiler module in Chenomx with the integrated 1D spectral library from the Human Metabolome Database (HMDB). The resulting metabolite concentration matrix was exported for subsequent multivariate, pathway enrichment, and correlation analyses [27].

### 2.5. Multivariate Metabolomic Analysis

^1^H NMR metabolomic data were analyzed using MetaboAnalyst 6.0 (Xia Lab, McGill University, Montreal, QC, Canada) and R (version 4.3.1, R Core Team, Vienna, Austria) using the mixOmics package [28]. Prior to statistical analyses, data were subjected to a standardized preprocessing workflow: missing values were replaced with half of the minimum positive value in the dataset, no sample normalization was applied (None), and values were log-transformed (Log_10_) and scaled using auto-scaling. An unsupervised Principal Component Analysis (PCA) was initially performed using all ruminal metabolomic profiles to provide an overview of the global metabolic variation across sampling times (day 0 and day 21). Subsequently, supervised Partial Least Squares–Discriminant Analysis (PLS-DA) was performed separately for each experimental treatment (CTL, EE, HE, and MON) to exploratively assess treatment-specific temporal patterns in the ruminal metabolome by comparing samples collected before supplementation (day 0) with those collected after 21 days of supplementation.

Model performance was assessed by cross-validation using R^2^X, R^2^Y, and Q^2^, and permutation testing was performed using both the classification error rate (CER) and Q^2^ to assess model discrimination and predictive performance, respectively. Metabolites contributing to class discrimination were identified according to their Variable Importance in Projection (VIP) scores. Statistical differences in subclass proportions between day 0 and day 21 were assessed using paired comparisons within treatment. Normality of the within-animal differences was assessed using the Shapiro–Wilk test. When normality assumptions were met, a paired Student’s *t*-test was applied; otherwise, the Wilcoxon signed-rank test was used. Raw *p*-values were adjusted for multiple testing using the Benjamini–Hochberg false discovery rate (FDR), and significance was declared at adjusted *p* < 0.05.

### 2.6. Chemical Taxonomy and Subclass Classification

To evaluate structural shifts in the ruminal metabolome, identified metabolites were classified into chemical macro-classes and subclasses according to the ChemONT taxonomy using the Bovine Metabolome Database (https://bovinedb.ca/ (accessed on 8 May 2026)) [29]. Because only the EE treatment showed evidence of treatment-specific temporal separation in the PLS-DA analysis, subclass comparisons were restricted to the EE group and were therefore considered exploratory. The relative contribution (%) of each chemical subclass to the total profiled metabolome was calculated for each sampling point. Statistical differences in subclass proportions between day 0 and day 21 were assessed for each subclass individually. Normality of the within-animal differences was assessed using the Shapiro–Wilk test. When normality was met, paired Student’s *t*-tests were applied; otherwise, the Wilcoxon signed-rank test was used. Raw *p*-values were adjusted for multiple testing using the Benjamini–Hochberg false discovery rate (FDR), and statistical significance was declared at adjusted *p* < 0.05.

### 2.7. Correlation Analysis

Because only the EE treatment showed evidence of treatment-specific temporal metabolomic separation in the PLS-DA analysis, subsequent correlation analyses were restricted to the EE group and were considered exploratory. Associations between ruminal metabolites, ruminal fermentation variables, and archaeal taxa were evaluated using Spearman’s rank correlation coefficient. Correlation analyses were performed separately for (i) ruminal fermentation variables vs. archaeal relative abundances and (ii) ruminal fermentation variables vs. metabolite concentrations. Nominal statistical significance was defined as *p* < 0.05, whereas false discovery rate (FDR)-adjusted *p*-values were also calculated using the Benjamini–Hochberg procedure to account for multiple testing [30]. Correlation matrices were generated and visualized in R using the packages corrplot and ComplexHeatmap.

### 2.8. Statistical Analysis

Ruminal fermentation parameters (pH, NH_3_-N, and SCFA), archaeal alpha diversity indices, and individual metabolite concentrations were analyzed using the GLIMMIX procedure of SAS (version 9.4, SAS Institute Inc., Cary, NC, USA). Residual normality was assessed using the Shapiro–Wilk test through UNIVARIATE procedure, and extreme outliers (exceeding ±3 standard deviations) were checked and removed from the dataset whenever detected.

The statistical model used for these variables was:

Yijkl = μ + Ti + Sj + Pk + A(S)jl + eijkl

where Yijkl: dependent variable; µ: overall mean; Ti: fixed effects of treatment (i = 1, 2, 3, 4); Sj: random effect of square (j = 1, 2); Pk: random effect of the period (k = 1, 2, 3, 4); A(S)jl: random effect of animal nested within square (l = 1, 2, 3, 4, 5, 6, 7, 8); and eijkl: the experimental error.

Least squares means were calculated using the LSMEANS statement. Specific biological hypotheses were tested using pre-planned orthogonal contrasts:

1. Additive effects (C1): control vs. the average of all additive treatments [CTL vs. (EE + HE + MON)/3];
2. Extracts vs. monensin (C2): the average of plant extracts vs. monensin [(EE + HE)/2 vs. MON];
3. Extract type (C3): ethanolic extract vs. hydroethanolic extract (EE vs. HE).

Least squares means were compared using Tukey’s test when appropriate. Additionally, to account for multiple testing among archaeal genera, *p*-values for relative abundances were adjusted using the Bonferroni correction, with adjusted *p* < 0.05 considered statistically significant. Differences were considered significant at *p* < 0.05, and trends were discussed when 0.05 < *p* ≤ 0.10. It is important to note that these tendencies are based on nominal, uncorrected *p*-values. Given the multiple pre-planned orthogonal contrasts evaluated across different variables, these trends should be interpreted with caution. The individual animal was considered the experimental unit for all analyses.

Statistical analyses of the untargeted metabolomic data were performed separately, as described in Section 2.5, Section 2.6 and Section 2.7.

## 3. Results

### 3.1. Rumen Fermentation Parameters

Dry matter intake (DMI), ruminal pH, NH_3_-N and total SCFA concentration, and the molar proportions of acetate, isobutyrate, and butyrate were not affected by supplementation with *U. brizantha* extracts or monensin (*p* > 0.10; Table 1).

Despite the absence of main effects, orthogonal contrast analysis revealed specific trends in ruminal fermentation. Regarding nitrogen metabolism, NH_3_-N concentrations tended to be lower in animals receiving MON compared to those supplemented with plant extracts (C2; *p* = 0.093). Additionally, ruminal NH_3_-N levels tended to be higher in the EE compared to the HE groups (C3; *p* = 0.083).

Shifts in the SCFA profile were also observed as marginal trends. Monensin supplementation tended to increase propionate proportions compared to the plant extracts (C2; *p* = 0.067). When comparing the control group against all additives (C1), a tendency for higher isovalerate proportions (*p* = 0.062) and a higher acetate:propionate ratio (*p* = 0.094) was detected in supplemented animals. Finally, valerate proportions were also influenced by the type of extract, tending to be higher in sheep receiving EE (1.45%) compared to those receiving HE (1.11%) (C3; *p* = 0.070).

### 3.2. Methanogenic Archaeal Diversity and Relative Abundance

After sequence quality control, a total of 5,132,174 reads were retained, resulting in 8166 ASVs used for downstream microbiome analyses. The average sequencing depth was 81,463 reads per sample. Taxonomic assignment classified 301 ASVs at the kingdom level, 285 at the phylum level, 282 at the class level, 282 at the order level, 272 at the family level, and 248 at the genus level, whereas no ASVs were classified at the species level. This lack of species-level classification reflects the resolution limitation of targeting a short single hypervariable region (~315 bp V8 amplicon) compared to full-length 16S rRNA gene or metagenomic approaches.

The effects of EE, HE, and MON on archaeal alpha diversity and relative abundance are shown in Table 2. No overall treatment effect was detected for alpha diversity indices (*p* > 0.175). However, orthogonal contrast C2 showed lower Shannon diversity in sheep supplemented with *U. brizantha* extracts than in those receiving monensin (*p* = 0.034), while Simpson diversity showed a tendency (*p* = 0.065) in the same direction.

At the genus level, *Methanimicrococcus* was the only genus showing a significant treatment effect (*p* = 0.012, P_adj_ = 0.048 after Bonferroni correction across genera), showing greater relative abundance in EE (1.63%) than in CTL (0.46%), HE (0.60%), and MON (0.07%). In contrast, the dominant hydrogenotrophic genus *Methanobrevibacter*, as well as *Methanosphaera* and *Methanoplanus*, showed no detectable treatment-associated differences (*p* > 0.05). Although *Methanoplanus* was displayed as <0.01% in Table 2 because of its very low abundance, statistical analyses were performed using the unrounded relative-abundance values, yielding no detectable treatment effect (*p* = 0.686).

### 3.3. Rumen Untargeted Metabolomics via ^1^H NMR

**Figure 1:**
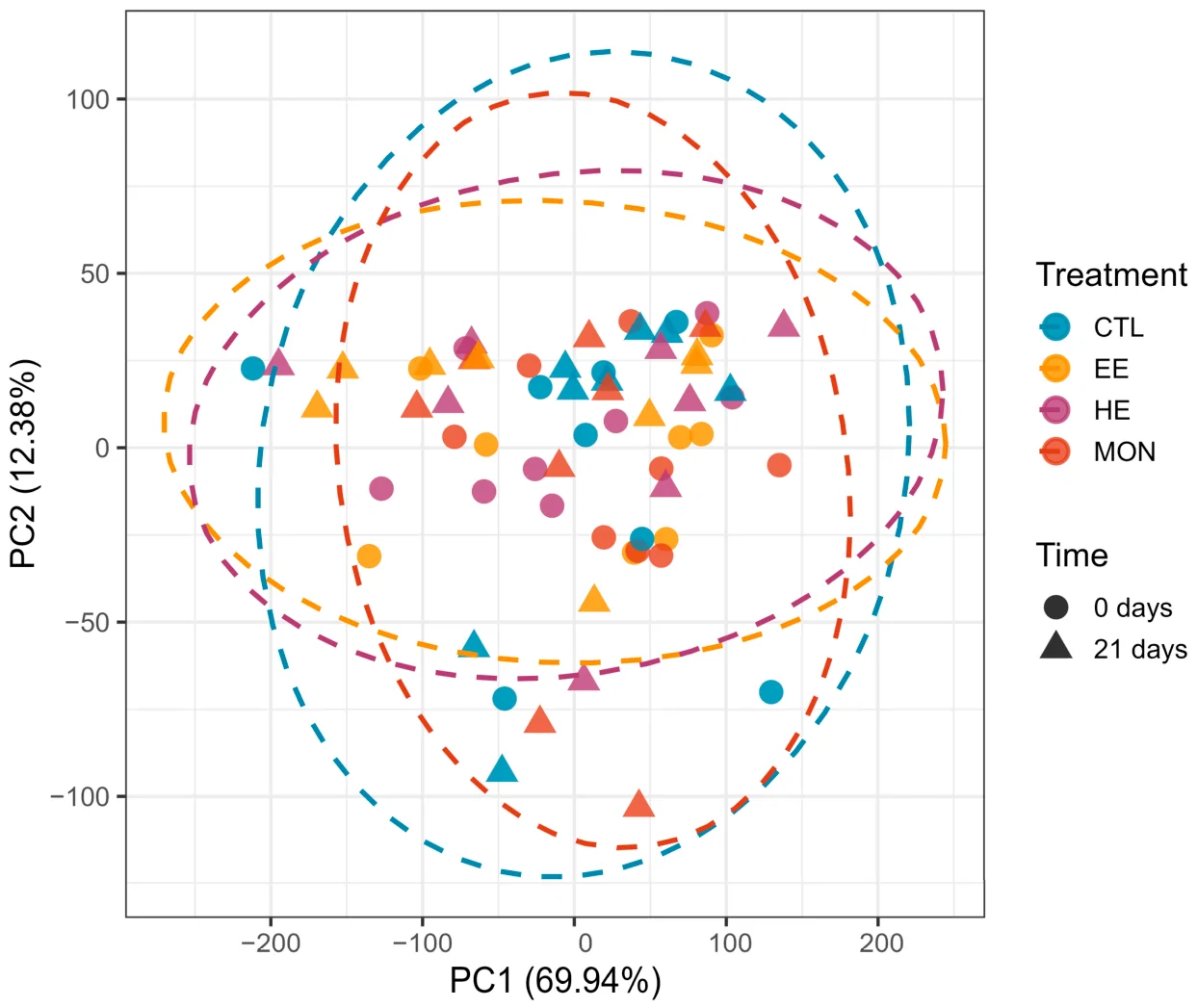
Principal Component Analysis (PCA) of the ruminal fluid metabolome from 64 samples obtained from eight sheep across four treatments and two sampling times (day 0 and day 21). Treatments are color-coded (CTL, EE, HE, MON), and sampling day is indicated by symbol shape.

Multivariate PCA and PLS-DA analyses were employed in a strictly exploratory capacity to visualize global temporal trajectories and metabolomic profile separation between baseline (day 0) and day 21 across treatments. Unsupervised PCA of all ruminal metabolomic profiles revealed substantial overlap between sampling times, with the first two principal components explaining 82.32% of the total metabolomic variance (PC1 = 69.94%; PC2 = 12.38%; Figure 1).

**Figure 2:**
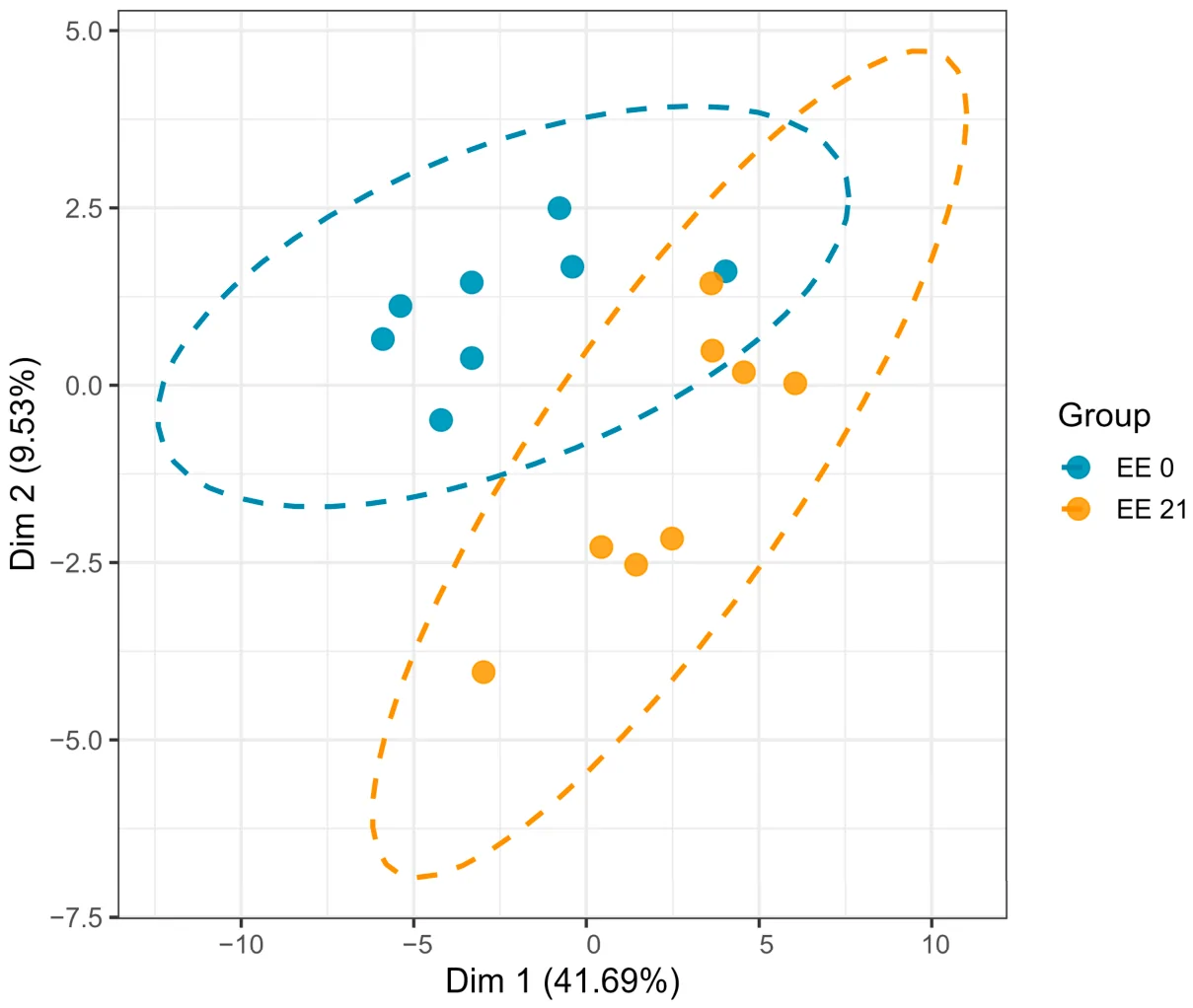
Partial Least Squares–Discriminant Analysis (PLS-DA) score plot showing the treatment-specific temporal separation of ruminal metabolomic profiles within the ethanolic extract (EE) treatment between baseline (day 0) and day 21. Four-component model: R^2^X = 0.713, R^2^Y = 0.921, Q^2^ = 0.504; CER = 0.2031; CER permutation test, *p* = 0.027; Q^2^ permutation test, *p* = 0.058; 2000 permutations; *n* = 8.

The exploratory PLS-DA indicated a temporal pattern in the EE group, with partial separation between day 0 and day 21 samples (Figure 2). The four-component EE model accounted for substantial variance (R^2^X = 0.713, R^2^Y = 0.921), with moderate cross-validated predictability (Q^2^ = 0.504). The classification error rate (CER) was 0.184, and the CER permutation test indicated better classification performance than expected by permutation (*p* = 0.027). However, the Q^2^ permutation test was not significant (*p* = 0.058), indicating limited evidence for robust predictive separation. Therefore, the temporal separation observed in the EE group was interpreted as exploratory rather than confirmatory. In contrast, no significant temporal discrimination was detected for the CTL, HE, or MON treatments (*p* > 0.05).

**Figure 3:**
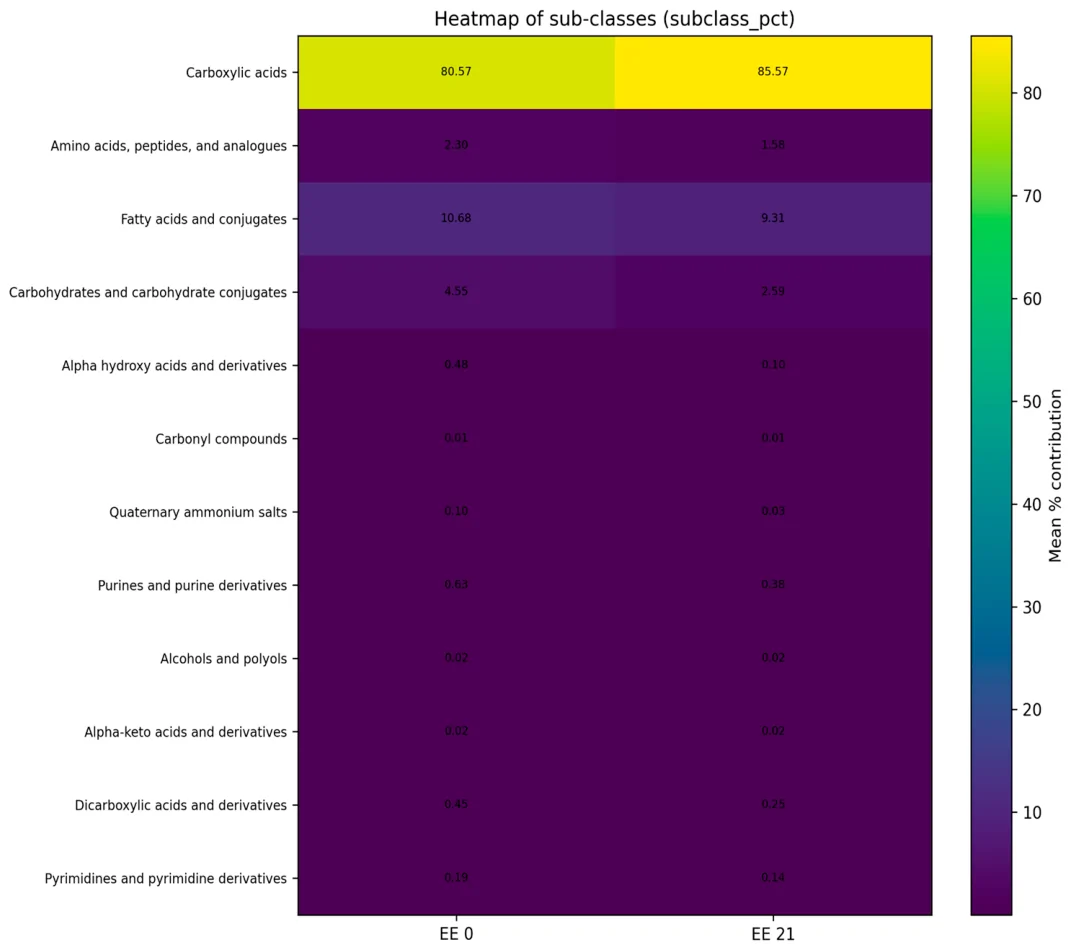
Chemical subclass proportions in the ruminal metabolome of sheep in the EE group (day 0 and day 21). Values represent mean percentage contribution (%). EE, ethanolic extract (*n* = 8).

Chemical taxonomy classification revealed structural shifts in the ruminal metabolome of EE-supplemented animals at the subclass level (Figure 3). Carboxylic acids represented the dominant fraction of the profiled metabolome and increased significantly from baseline (80.57% vs. 85.57%; *p* = 0.002, FDR = 0.022). Conversely, several minor subclasses displayed numerical reduction, including carbohydrates and carbohydrate conjugates (4.55% vs. 2.59%; *p* = 0.083, FDR = 0.222), dicarboxylic acids and derivatives (0.45% vs. 0.25%; *p* = 0.0881, FDR = 0.222), and amino acids, peptides, and analogues (2.30% vs. 1.58%; *p* = 0.0988, FDR = 0.222). Additional subclasses, including alpha hydroxy acids and derivatives (0.48% vs. 0.10%; *p* = 0.1049), purines and purine derivatives (0.63% vs. 0.38%; *p* = 0.1298), and quaternary ammonium salts (0.10% vs. 0.03%; *p* = 0.1304), also exhibited numerical decreases (FDR > 0.05).

Variable Importance in Projection (VIP) highlighted the highest-ranking metabolites contributing to the observed temporal separation within the EE group between day 0 and day 21. Aspartate (VIP = 1.91), succinate (VIP = 1.82), glutamate (VIP = 1.79), phenylalanine (VIP = 1.73), threonine (VIP = 1.53), and leucine (VIP = 1.45) were the principal drivers of the discrimination between day 0 and day 21 samples.

**Figure 4:**
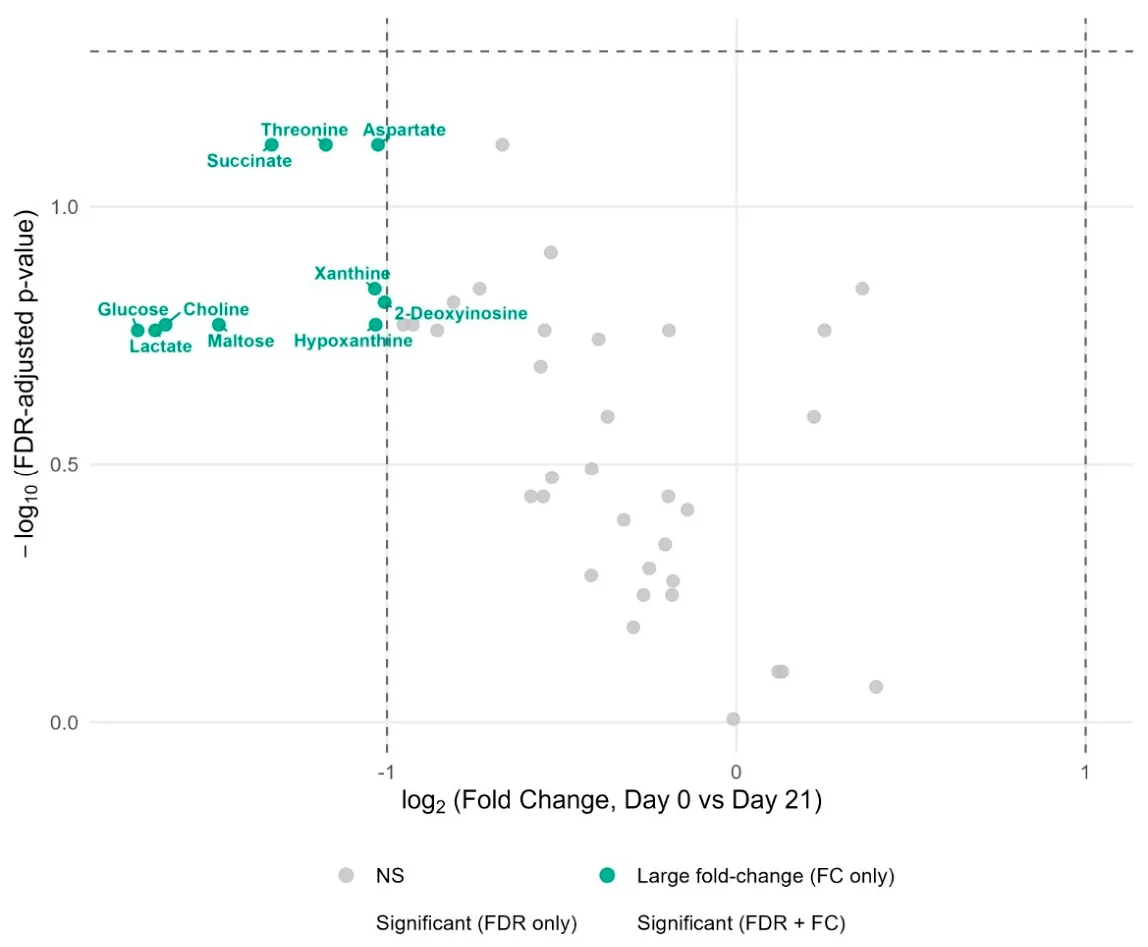
Volcano plot of ruminal metabolite alterations in sheep receiving the EE between baseline (day 0) and day 21 (*n* = 8). Green points indicate features with large fold-change alone (|log_2_FC| ≥ 1.0; x-axis), while grey points represent non-significant metabolites (NS). Dashed lines mark the cutoffs for fold-change and statistical significance (−log_10_ FDR < 0.05; y-axis). No metabolite reached combined FDR and FC significance.

Compared to baseline values (day 0), EE supplementation exhibited reductions in several amino acids and fermentation intermediate pools by day 21 (Figure 4). Nominal decreases (raw *p* < 0.01, FDR = 0.0602) were observed for aspartate (84.91 ± 8.67 vs. 42.94 ± 6.30; *p* = 0.0018, −49.4%), succinate (101.34 ± 11.30 vs. 46.64 ± 10.33; *p* = 0.0031, −54.0%), glutamate (118.24 ± 7.18 vs. 69.74 ± 7.18; *p* = 0.0049, −41.0%), and phenylalanine (16.64 ± 1.41 vs. 10.34 ± 1.31; *p* = 0.0056, −37.9%). Further numerical decreases were observed for leucine (41.48 ± 6.28 vs. 22.25 ± 4.67; *p* = 0.0290, −46.4%) and choline (23.39 ± 5.69 vs. 7.99 ± 4.01; *p* = 0.0462, −65.8%) (FDR > 0.20).

The volcano plot constructed from the paired comparison between baseline (day 0) and day 21 of EE supplementation identified several metabolites exhibiting large numerical changes in abundance (|log_2_FC| ≥ 1.0; Figure 4). Although no individual metabolite remained statistically significant after Benjamini–Hochberg multiple-testing correction (FDR < 0.05), succinate (log_2_FC = −1.33, FDR = 0.076), threonine (log_2_FC = −1.18, FDR = 0.076), and aspartate (log_2_FC = −1.03, FDR = 0.076) showed the strongest statistical evidence of change. Several metabolites also displayed large numerical decreases, including glucose (log_2_FC = −1.71, FDR = 0.174), lactate (log_2_FC = −1.66, FDR = 0.174), choline (log_2_FC = −1.63, FDR = 0.169), maltose (log_2_FC = −1.48, FDR = 0.169), and 2-deoxyinosine (log_2_FC = −1.01, FDR = 0.153). Thus, while multiple metabolites exhibited large fold changes between day 0 and day 21, none met the predefined threshold for statistical significance after FDR correction.

To determine whether these temporal metabolic changes translated into differences among treatments at the end of the supplementation period, compounds were ranked by their mean absolute concentration (µM) on day 21, and the ten most abundant metabolites quantified by ^1^H NMR were compared among treatments (Table 3).

Although no main treatment effects were observed, orthogonal contrast C2 indicated that animals receiving MON tended to exhibit greater concentrations of glutamate (*p* = 0.056), maltose (*p* = 0.081), glucose (*p* = 0.088), and xanthine (*p* = 0.065) than animals supplemented with the plant extracts. In addition, orthogonal contrast C1 indicated that CTL animals tended to exhibit greater leucine concentrations than supplemented animals (*p* = 0.060). No differences were detected between EE and HE (C3; *p* > 0.414).

### 3.4. Correlation Between Metabolites, Ruminal Fermentation and Archaeal Community

Correlation analyses were performed within the EE treatment to investigate the relationships between: (i) ruminal fermentation parameters and archaeal genera; (ii) ruminal fermentation parameters and metabolite concentrations.

**Figure 5:**
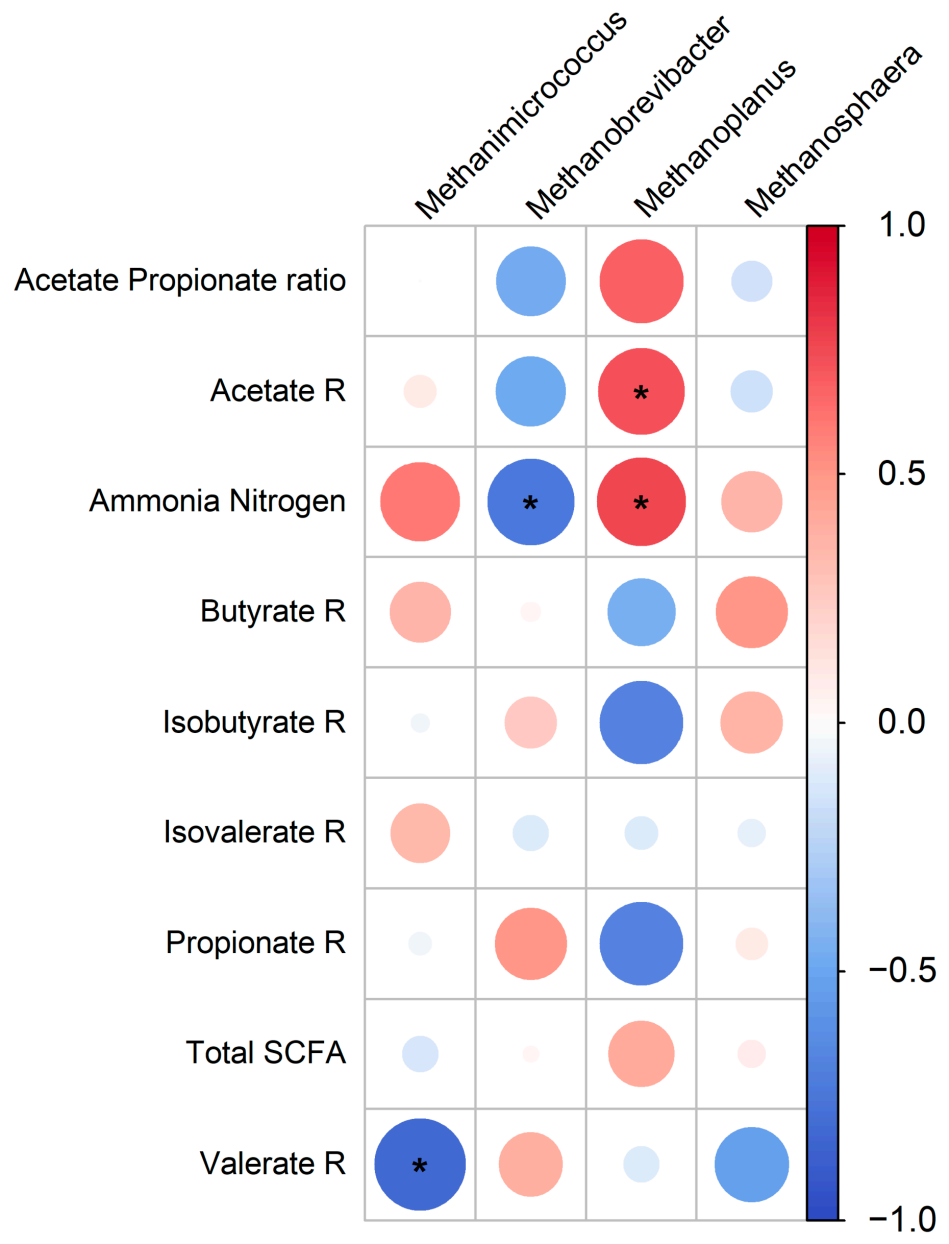
Correlation matrix between ruminal fermentation parameters and archaeal genera relative abundance in sheep supplemented with the ethanolic extract (EE). Circle size and color intensity represent the correlation coefficient (r) (red: positive, blue: negative). Asterisks (*) indicate nominal significance (uncorrected *p* < 0.05). No correlations remained significant after False Discovery Rate (FDR) correction.

The phenotype–archaeal correlation matrix highlighted several significant associations between ruminal fermentation parameters and archaeal genera. NH_3_-N was negatively correlated with *Methanobrevibacter* (r = −0.74, *p* = 0.046) and positively correlated with *Methanoplanus* (r = 0.77, *p* = 0.027), consistent with potential relationships between nitrogen metabolism and these methanogenic taxa. Valerate proportion showed a strong negative correlation with *Methanimicrococcus* (r = −0.81, *p* = 0.027). In addition, acetate proportion was positively associated with *Methanoplanus* (r = 0.73, *p* = 0.041), whereas propionate proportion tended to negatively correlate with this genus (r = −0.68, *p* = 0.065) (Figure 5).

**Figure 6:**
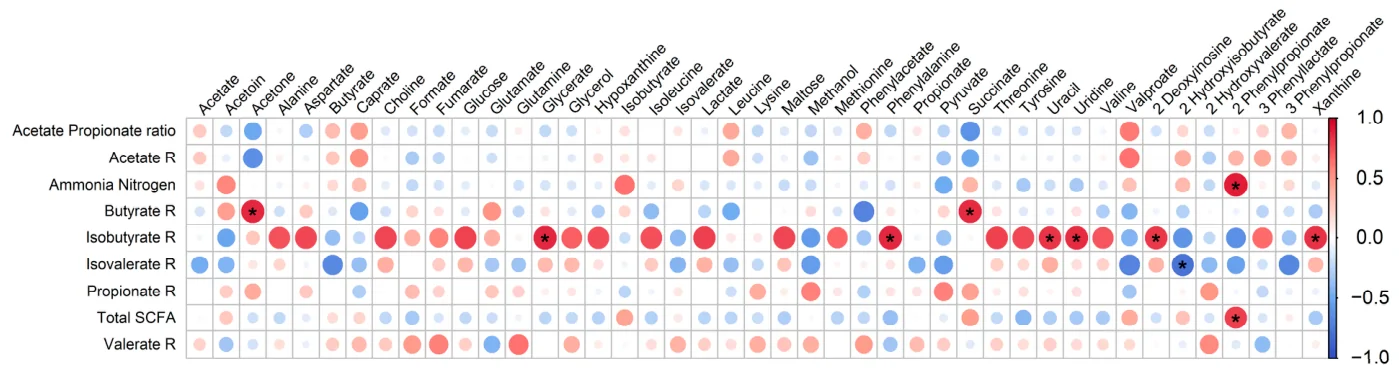
Correlation matrix between ruminal fermentation parameters and ruminal metabolites detected by ^1^H NMR in sheep supplemented with the ethanolic extract (EE). Circle size and color intensity represent the correlation coefficient (r) (red: positive, blue: negative). Asterisks (*) indicate nominal significance (uncorrected *p* < 0.05). No correlations remained significant after False Discovery Rate (FDR) correction.

The phenotype–metabolite correlation analysis identified several strong associations between ruminal fermentation traits and individual metabolite concentrations (Figure 6). Ammonia nitrogen was positively correlated with 2-phenylpropionate (r = 0.89, *p* = 0.003), whereas butyrate showed positive correlations with acetone (r = 0.87, *p* = 0.011) and succinate (r = 0.86, *p* = 0.013). Isobutyrate was also positively associated with several amino acid- and nucleotide-related metabolites, including phenylalanine (r = 0.86, *p* = 0.026), glycerate (r = 0.86, *p* = 0.028), uridine (r = 0.85, *p* = 0.030), xanthine (r = 0.84, *p* = 0.038), and uracil (r = 0.82, *p* = 0.044). Conversely, isovalerate was negatively correlated with 2-hydroxyisobutyrate (r = −0.78, *p* = 0.022).

## 4. Discussion

A key finding of the present study was the marked dissociation between conventional ruminal fermentation measurements and the functional responses revealed by the multi-omics approach. Although DMI, ruminal pH, NH_3_-N, and the major SCFAs remained largely unchanged, metabolomic profiling and archaeal community analysis revealed clear extract-specific biological responses. These responses should be interpreted in the context of the conservative supplementation levels evaluated. The dietary concentrations of protodioscin, the principal steroidal saponin supplied by the extracts, ranged from 0.375 to 0.598 g/kg DM, substantially below the saponin concentrations commonly investigated for methane mitigation in vivo (up to 5.0 g/kg DM) [31,32]. The preservation of conventional fermentation parameters, despite these extract-specific responses, suggests that *U. brizantha* secondary metabolites influenced intracellular metabolic processes and microbial interactions without substantially disrupting overall fermentative function. This pattern is consistent with functional redundancy, whereby distinct microbial communities maintain similar ecosystem-level outputs despite shifts in taxonomic composition, metabolic interactions, or intracellular pathways [11,33,34,35].

Among archaeal populations, the most prominent response was the selective enrichment of the methylotrophic genus *Methanimicrococcus* in animals supplemented with the ethanolic extract. In contrast, no treatment-associated differences were detected for the dominant hydrogenotrophic genus *Methanobrevibacter*, which remained predominant across all treatments, whereas *Methanoplanus* occurred at very low relative abundance throughout the experiment. Similar patterns have been reported following supplementation with steroidal saponins, where methanogenic communities generally exhibit subtle compositional adjustments instead of extensive taxonomic reorganization [36,37]. The persistence of *Methanobrevibacter* as the predominant genus is also consistent with previous observations in forage-fed cattle, reinforcing its central ecological role in ruminal archaeal communities despite moderate dietary interventions [38,39].

The *Methanimicrococcus* response may be interpreted in light of its known metabolic specialization. Unlike hydrogenotrophic methanogens, *Methanimicrococcus* lacks the complete Wood–Ljungdahl pathway and therefore depends almost exclusively on methylated compounds rather than CO_2_/H_2_ for methanogenesis [40]. Although this genus typically represents only a minor fraction of the ruminal archaeome, particularly in feedlot systems (0.01% to 0.05%) [25,41], forage-based diets provide greater availability of pectin-derived methyl compounds and may therefore support methylotrophic metabolism through enhanced microbial cross-feeding [42,43,44]. Thus, the selective enrichment observed here may reflect changes in substrate availability within a specialized ecological niche rather than generalized stimulation of methanogenesis. During microbial degradation of pectin-rich plant cell walls, bacterial fermentation releases methanol, while choline metabolism generates methylamines, both of which constitute major substrates for methylotrophic methanogenesis [45]. Interestingly, despite the selective enrichment of *Methanimicrococcus*, ruminal methanol concentration remained unchanged among treatments, suggesting that substrate production and microbial utilization may have remained closely balanced, resulting in little net accumulation in ruminal fluid.

The contrasting responses observed between EE and HE further highlight the potential influence of extraction methodology; however, it is crucial to note that this comparison is heavily confounded by the unequal daily delivery of saponin mass (375 vs. 598.4 mg/day of protodioscin for EE and HE, respectively). Although solvent-dependent variation has been consistently reported for saponin-rich botanical extracts [6,36], the differences observed in the present study cannot be solely attributed to the solvent polarity because extraction method and saponin dose differed simultaneously. Pure ethanol preferentially extracts relatively less polar and more lipophilic secondary metabolites, whereas hydroethanolic mixtures recover a broader spectrum of polar phytochemicals [46,47]. Although the complete phytochemical composition of each extract was not determined in the present study, these physicochemical differences likely generated distinct biochemical environments within the rumen, thereby contributing to the divergent archaeal responses observed between extraction methods.

Although direct evidence linking steroidal saponins specifically to methylotrophic methanogens remains scarce, recent advances in ruminal microbiology provide a plausible ecological framework for interpreting these findings. Despite the selective enrichment of *Methanimicrococcus*, ruminal methanol concentrations remained unchanged among treatments, potentially reflecting rapid substrate turnover and a balance between methanol production and microbial utilization. Untargeted metabolomics further identified a numerical shift in choline metabolism based on the fold-change criterion (|log_2_FC| ≥ 1.0; log_2_FC = −1.63, FDR = 0.169) in EE-supplemented animals, although these did not retain strict significance after multiple-testing correction (FDR > 0.05). Because microbial degradation of choline generates mono-, di-, and trimethylamine, preferential substrates for methylotrophic methanogens [48,49], this finding is consistent with the possibility that EE supplementation was associated with changes in methyl group-related metabolism and substrate availability for *Methanimicrococcus.* However, because methylamine concentrations and metabolic fluxes were not directly quantified, this proposed mechanism remains speculative.

These functional relationships were further explored through phenotype–archaeal correlation analyses. Although none of the observed associations remained significant after false discovery rate correction (FDR > 0.05), several were biologically coherent and therefore useful for hypothesis generation. *Methanoplanus* was positively associated with acetate and NH_3_-N and negatively associated with propionate, consistent with the metabolic relationships underlying hydrogenotrophic methanogenesis and SCFA production [39]. Conversely, the negative association between *Methanimicrococcus* and valerate may reflect its distinct methylotrophic metabolism rather than a direct relationship with conventional SCFA production. Similarly, the negative association between isovalerate and 2-hydroxyisobutyrate may indicate localized metabolic partitioning during branched-chain amino acid metabolism [48,49]. Given the exploratory nature of these correlations, these associations should be interpreted as hypothesis-generating rather than confirmatory.

Beyond archaeal niche specialization, untargeted metabolomics revealed coordinated shifts in metabolites related to central carbon and nitrogen metabolism in EE-supplemented animals. The marked reductions in glutamate and aspartate are particularly informative because these metabolites occupy central positions in microbial nitrogen assimilation, transamination reactions, and amino acid biosynthesis [50,51]. Importantly, these metabolic changes occurred without detectable alterations in ruminal NH_3_-N concentration, indicating that ammonia availability remained balanced despite substantial intracellular nitrogen metabolism. Rather than indicating impaired proteolysis, depletion of these intracellular amino acid pools may reflect enhanced microbial utilization of preformed nitrogen compounds for anabolic processes, particularly microbial protein synthesis [52]. This interpretation is consistent with the dynamic nature of ruminal nitrogen metabolism, in which extracellular NH_3_-N represents the net balance among protein degradation, amino acid deamination, microbial uptake, and nitrogen recycling rather than a direct measure of any individual process.

This interpretation is further supported by the exploratory phenotype–metabolite associations. Although these pairwise relationships did not remain significant following FDR correction (FDR > 0.05), their biological coherence provides additional context for the observed metabolic changes. NH_3_-N showed positive alignment with 2-phenylpropionate, a product of aromatic amino acid degradation, whereas isobutyrate was associated with amino acids and nucleotide degradation intermediates. Together, these associations are consistent with ongoing microbial turnover and nitrogen cycling, while the stability of NH_3_-N may reflect rapid metabolic exchange rather than metabolic inactivity.

Another notable metabolic response was the substantial reduction (−54%) in ruminal succinate following EE supplementation. Succinate represents one of the principal intermediates linking cellulolytic fermentation to propionate production and is continuously exchanged among microbial populations through metabolic cross-feeding [53,54]. Consequently, decreases in free succinate concentration should not necessarily be interpreted as reduced fermentative activity. Instead, lower standing concentrations may indicate more rapid microbial turnover of this intermediate, resulting in tighter coupling between succinate-producing and succinate-consuming populations.

This interpretation is fully consistent with the absence of changes in total SCFA concentrations. Because ruminal metabolomics captures metabolite pools at a single sampling point rather than metabolic fluxes, depletion of an intermediate metabolite may reflect increased metabolic throughput instead of reduced pathway activity. Accordingly, the reduction in succinate together with stable propionate concentrations may indicate subtle changes in carbon partitioning without major alterations in end-product fermentation. Nominal associations among butyrate, succinate, and ketone-related metabolites, including acetone, further support the coordinated nature of these metabolic changes, although these relationships were exploratory and did not remain significant after FDR correction. Together, these findings suggest that EE primarily influenced intermediary metabolic organization rather than overall fermentative capacity.

Comparison with monensin further highlights the distinct biological behavior of the phytogenic extracts. Whereas EE treatment selectively increased *Methanimicrococcus* abundance, MON treatment maintained this genus at levels comparable to the CTL. Likewise, the absence of enrichment in the HE treatment agrees with observations reported by [25] using hydroethanolic walnut husk extracts, suggesting that extraction methodology is an important determinant of phytogenic activity. From a mechanistic perspective, these contrasting responses suggest different response patterns under the present experimental conditions. Monensin acts primarily through a broad antimicrobial mechanism, selectively inhibiting Gram-positive bacteria, altering transmembrane ion gradients, and indirectly reducing hydrogen availability for methanogenesis [2,54]. In contrast, the ethanolic *U. brizantha* extract produced little evidence of widespread microbial disruption. Instead, the combined metabolomic and archaeal observations are more consistent with selective metabolic responses than with broad microbial disruption, although direct mechanistic inference was beyond the scope of the study, characterized by changes in precursor availability, intermediary metabolism, and specialized microbial niches. This pattern is more consistent with selective metabolic modulation within an otherwise stable ruminal ecosystem than with a classical antimicrobial effect.

Among these phytochemicals present in *Urochloa* species, steroidal saponins represent plausible candidates for the metabolic responses observed in the present study. These amphiphilic molecules, including protodioscin, possess a lipophilic steroidal aglycone linked to hydrophilic sugar moieties, enabling selective interactions with microbial cell membranes and potentially modifying microbial metabolism without extensive disruption of community structure [15,33]. Previous studies using steroidal saponins from *Yucca schidigera* and triterpenoid saponins from *Quillaja saponaria* indicate that their biological responses depend strongly on chemical structure, botanical origin, extraction methodology, and supplementation level [6,8,32,36]. The protodioscin concentrations supplied in the present study (0.375–0.598 g/kg DM) were considerably lower than those commonly evaluated for methane mitigation in vivo (up to 5 g/kg DM) [31,32]. Therefore, the relatively modest microbial responses observed here may reflect the conservative supplementation rather than limited biological activity. As one of the first in vivo evaluations of *U. brizantha* extracts, the present findings should be viewed as preliminary in vivo evidence of selective archaeal and metabolomic responses to support future dose–response investigations rather than defining the maximal bioactive potential of these extracts. Taken together, these findings indicate that metabolomic and archaeal profiling can reveal functional changes that are not apparent from conventional fermentation measurements, highlighting the capacity of the ruminal ecosystem to maintain overall fermentative stability despite metabolic reorganization [11,33,34,35].

Several limitations should nevertheless be acknowledged. First, ruminal fermentation parameters were evaluated at a single time point (4 h post-feeding), selected to synchronize fermentation measurements with the multi-omics analyses but unable to capture full diurnal kinetics or nadir responses. Second, the relatively conservative protodioscin inclusion levels limited assessment of the maximal biological response achievable with *U. brizantha* extracts. Third, the comparison between the ethanolic and hydroethanolic extracts is inherently confounded by differences in daily protodioscin dose (375.0 vs. 598.4 mg/day for EE and HE, respectively), because extract volumes rather than protodioscin content were standardized; therefore, differences between EE and HE cannot be attributed exclusively to extraction solvent polarity. Fourth, interpretation of the phenotype–metabolite and phenotype–archaeal associations should remain cautious because these exploratory associations were not significant after false discovery rate correction (FDR > 0.05). Fifth, archaeal profiling was based on a short (~315 bp) V8 16S rRNA amplicon, limiting taxonomic resolution compared with full-length 16S rRNA sequencing or shotgun metagenomics and precluding confident species-level assignment. Finally, although the metabolomic profiles were consistent with potential alterations in methyl-group metabolism and microbial cross-feeding, direct quantification of methylamines and metabolic fluxes was beyond the scope of the present study. Future studies integrating stable isotope tracing, targeted metabolomics, metatranscriptomics, or fluxomics could provide valuable confirmation of the metabolic pathways proposed here.

Despite these limitations, the present study adds preliminary in vivo evidence that *U. brizantha* extracts may be associated with specific metabolic and archaeal shifts in the rumen, even when conventional fermentation endpoints remain unchanged. Rather than indicating broad antimicrobial effects, the ethanolic extract was associated with differences in intermediary carbon and nitrogen metabolism together with selective shifts in methylotrophic archaeal populations. These findings highlight the value of multi-omics approaches for detecting response patterns that may not be captured by conventional ruminal fermentation measurements and warrant further investigation into the potential of phytogenic additives to modulate ruminal function.

## 5. Conclusions

The ethanolic extract was associated with differences in intermediary carbon and nitrogen metabolism together with a relative enrichment of the methylotrophic archae on *Methanimicrococcus*, suggesting a pattern more consistent with targeted biological responses than with broad microbial disruption. Because the experimental treatments differed simultaneously in solvent extraction, administered volume, and protodioscin dose, the specific contribution of extraction polarity to phytochemical bioactivity remains to be determined in future matched-dose studies. More broadly, these findings highlight the value of integrating metabolomics with microbial community profiling to detect response patterns that may not be evident from conventional fermentation measurements alone. Overall, this study provides a basis for future dose–response and in vivo validation studies evaluating the potential of *U. brizantha*-derived phytogenic additives to influence ruminal function.

## Figures and Tables

**Table 1:** Ruminal fermentation parameters in sheep supplemented with *Urochloa brizantha* extracts or monensin in a replicated Latin square design (*n* = 8).

Items	Treatments *				SEM	*p*-Value			
	CTL	EE	HE	MON		Treat.	^1^ C1	^2^ C2	^3^ C3
DMI (kg)	0.94	0.74	0.85	0.86	0.10	0.439	0.187	0.507	0.358
pH	6.31	6.48	6.27	6.32	0.16	0.453	0.634	0.651	0.146
NH_3_-N (mg/dL)	5.75	6.37	5.09	4.62	0.92	0.097	0.487	0.093	0.083
SCFA (mM)	66.04	50.82	57.39	62.16	15.17	0.673	0.390	0.479	0.615
Acetate (%)	64.91	66.74	66.01	64.19	4.90	0.247	0.720	0.319	0.770
Propionate (%)	18.56	15.64	17.88	20.45	3.58	0.216	0.751	0.067	0.324
Isobutyrate (%)	1.74	2.27	1.89	1.77	0.46	0.359	0.369	0.248	0.257
Butyrate (%)	10.86	9.53	9.63	9.39	1.70	0.473	0.127	0.836	0.925
Isovalerate (%)	2.17	4.02	3.09	2.85	0.73	0.108	0.062	0.269	0.212
Valerate (%)	1.52	1.45	1.11	1.35	0.31	0.139	0.141	0.643	0.070
Acetate:Propionate ratio	2.99	5.20	4.99	4.00	1.46	0.269	0.094	0.307	0.861

* CTL: control; EE: ethanolic extract (50 mL/d); HE: hydroethanolic extract (80 mL/d); and MON: monensin (25 mg/kg of DMI); SEM: standard error of the mean. DMI: dry matter intake, NH_3_-N: ammonia nitrogen; SCFA: short-chain fatty acid. Orthogonal contrasts: ^1^ C1 = CTL vs. (EE + HE + MON)/3; ^2^ C2 = (EE + HE)/2 vs. MON; ^3^ C3 = EE vs. HE.

**Table 2:** Alpha diversity indices and relative abundance (%) of ruminal methanogenic archaea in sheep supplemented with *Urochloa brizantha* extracts or monensin (*n* = 8).

Items	Treatments *				SEM		*p*-Value		
	CTL	EE	HE	MON		Treat.	^1^ C1	^2^ C2	^3^ C3
Diversity indices									
Chao1	121.64	117.50	113.89	123.61	8.32	0.718	0.667	0.332	0.684
Shannon	2.43	2.30	2.39	2.67	0.13	0.175	0.877	0.034	0.567
Simpson	0.874	0.831	0.864	0.899	0.022	0.188	0.694	0.065	0.270
Relative abundance (%)									
*Methanobrevibacter*	92.80	92.64	84.25	87.72	6.20	0.494	0.412	0.901	0.198
*Methanosphaera*	1.06	0.60	1.07	1.16	0.22	0.371	0.684	0.296	0.176
*Methanimicrococcus*	0.46 ^b^	1.63 ^a^	0.60 ^b^	0.07 ^b^	0.51	0.012	0.505	0.009	0.038
*Methanoplanus* **	<0.01	<0.01	<0.01	<0.01	<0.01	0.686	0.386	0.640	0.503

* CTL: control; EE: ethanolic extract (50 mL/d); HE: hydroethanolic extract (80 mL/d); and MON: monensin (25 mg/kg of DMI); SEM: standard error of the mean. ** *Methanoplanus*: values were modeled using the unrounded relative-abundance data; values are shown as <0.01% because of their low magnitude. LSMEANS (and SEM) were: CTL = 0.0017%, EE = 0.0056%, HE = 0.0035%, and MON = 0.0032% (SEM = 0.0030%). ^a^, ^b^: Means within a row with different superscripts differ significantly by Tukey’s test (*p* < 0.05). Orthogonal contrasts: ^1^ C1 = CTL vs. (EE + HE + MON)/3; ^2^ C2 = (EE + HE)/2 vs. MON; ^3^ C3 = EE vs. HE.

**Table 3:** Concentrations of the ten most abundant metabolites (µM) in the rumen fluid of sheep supplemented with *Urochloa brizantha* extracts or monensin (*n* = 8).

Items	Treatments *				SEM	*p*-Value			
	CTL	EE	HE	MON		Treat.	^1^ C1	^2^ C2	^3^ C3
Amino acids									
Leucine	40.64	22.25	23.55	32.28	9.03	0.176	0.060	0.265	0.887
Alanine	114.59	75.42	92.04	116.47	37.77	0.232	0.278	0.118	0.453
Threonine	28.20	23.83	26.09	29.55	10.14	0.457	0.566	0.159	0.552
Glutamate	104.91	69.74	72.91	107.85	24.93	0.161	0.221	0.056	0.880
Carbohydrates									
Maltose	341.50	174.46	257.96	392.57	178.82	0.250	0.474	0.081	0.464
Glucose	560.59	287.84	359.38	607.41	304.15	0.267	0.354	0.088	0.702
Organic acids/intermediates									
Pyruvate	4.50	3.31	4.05	4.44	0.89	0.782	0.608	0.498	0.566
Glycerate	56.55	39.20	48.65	55.87	16.67	0.418	0.365	0.261	0.415
Propionate	4820.39	3657.64	4269.53	4463.07	521.65	0.468	0.261	0.441	0.414
Other metabolites									
Xanthine	76.35	62.42	59.25	77.36	34.55	0.165	0.203	0.065	0.746

* CTL: control; EE: ethanolic extract (50 mL/d); HE: hydroethanolic extract (80 mL/d); MON: monensin (25 mg/kg of DMI); SEM: standard error of the mean. Orthogonal contrasts: ^1^ C1 = CTL vs. (EE + HE + MON)/3; ^2^ C2 = (EE + HE)/2 vs. MON; ^3^ C3 = EE vs. HE.

## Data Availability

The data presented in this study are available from the corresponding author upon reasonable request.

## Abbreviations

The following abbreviations are used in this manuscript:

ADF	Acid Detergent Fiber
ASV	Amplicon Sequence Variant
CER	Classification Error Rate
CTL	Control
DM	Dry Matter
DMI	Dry Matter Intake
DSS	4,4-Dimethyl-4-silapentane-1-sulfonic acid
EE	Ethanolic Extract
FDR	False Discovery Rate
HE	Hydroethanolic Extract
HMDB	Human Metabolome Database
HPLC	High-Performance Liquid Chromatography
MON	Monensin
NDF	Neutral Detergent Fiber
NFC	Non-Fiber Carbohydrates
NH_3_-N	Ammonia Nitrogen
NMR	Nuclear Magnetic Resonance
PCA	Principal Component Analysis
PLE	Pressurized Liquid Extraction
PLS-DA	Partial Least Squares Discriminant Analysis
SCFA	Short-Chain Fatty Acid
VIP	Variable Importance in Projection
